# Posttreatment Attrition and Its Predictors, Attrition Bias, and Treatment Efficacy of the Anxiety Online Programs

**DOI:** 10.2196/jmir.3513

**Published:** 2014-10-14

**Authors:** Ali M AL-Asadi, Britt Klein, Denny Meyer

**Affiliations:** ^1^School of Health SciencesSwinburne University of TechnologyHawthornAustralia; ^2^Department of Arts and EducationGrande Prairie Regional CollegeGrande Prairie, ABCanada; ^3^DVC-Research & Innovation Portfolio; the School of Health Sciences and; the Collaborative Research NetworkFederation UniversityBallaratAustralia; ^4^National Institute of Mental Health ResearchThe Australian National UniversityCanberraAustralia; ^5^National eTherapy CentreSchool of Health SciencesSwinburne University of TechnologyHawthornAustralia

**Keywords:** posttreatment attrition, posttreatment predictors, treatment efficacy, online therapy, e-mental health, cognitive behavioral therapy, Internet interventions, fully automated, self-help, Web treatment, generalized anxiety disorder, obsessive compulsive disorder

## Abstract

**Background:**

Although relatively new, the field of e-mental health is becoming more popular with more attention given to researching its various aspects. However, there are many areas that still need further research, especially identifying attrition predictors at various phases of assessment and treatment delivery.

**Objective:**

The present study identified the predictors of posttreatment assessment completers based on 24 pre- and posttreatment demographic and personal variables and 1 treatment variable, their impact on attrition bias, and the efficacy of the 5 fully automated self-help anxiety treatment programs for generalized anxiety disorder (GAD), social anxiety disorder (SAD), panic disorder with or without agoraphobia (PD/A), obsessive-compulsive disorder (OCD), and posttraumatic stress disorder (PTSD).

**Methods:**

A complex algorithm was used to diagnose participants’ mental disorders based on the criteria of the Diagnostic and Statistical Manual of Mental Disorders (Fourth Edition, Text Revision; DSM-IV-TR). Those who received a primary or secondary diagnosis of 1 of 5 anxiety disorders were offered an online 12-week disorder-specific treatment program. A total of 3199 individuals did not formally drop out of the 12-week treatment cycle, whereas 142 individuals formally dropped out. However, only 347 participants who completed their treatment cycle also completed the posttreatment assessment measures. Based on these measures, predictors of attrition were identified and attrition bias was examined. The efficacy of the 5 treatment programs was assessed based on anxiety-specific severity scores and 5 additional treatment outcome measures.

**Results:**

On average, completers of posttreatment assessment measures were more likely to be seeking self-help online programs; have heard about the program from traditional media or from family and friends; were receiving mental health assistance; were more likely to learn best by reading, hearing and doing; had a lower pretreatment Kessler-6 total score; and were older in age. Predicted probabilities resulting from these attrition variables displayed no significant attrition bias using Heckman’s method and thus allowing for the use of completer analysis. Six treatment outcome measures (Kessler-6 total score, number of diagnosed disorders, self-confidence in managing mental health issues, quality of life, and the corresponding pre- and posttreatment severity for each program-specific anxiety disorder and for major depressive episode) were used to assess the efficacy of the 5 anxiety treatment programs. Repeated measures MANOVA revealed a significant multivariate time effect for all treatment outcome measures for each treatment program. Follow-up repeated measures ANOVAs revealed significant improvements on all 6 treatment outcome measures for GAD and PTSD, 5 treatment outcome measures were significant for SAD and PD/A, and 4 treatment outcome measures were significant for OCD.

**Conclusions:**

Results identified predictors of posttreatment assessment completers and provided further support for the efficacy of self-help online treatment programs for the 5 anxiety disorders.

**Trial Registration:**

Australian and New Zealand Clinical Trials Registry ACTRN121611000704998; http://www.anzctr.org.au/trial_view.aspx?ID=336143 (Archived by WebCite at http://www.webcitation.org/618r3wvOG).

## Introduction

In this age of technological advancement and the increase in peoples’ comfort in using the Internet and online resources, online therapy promises to provide an alternative methodology to face-to-face therapy and to be an effective vehicle to deliver treatment to individuals suffering from a variety of psychological disorders. The development and dissemination of e-mental health services have increased at an exponential rate [1]. This relatively new development has been progressing over the past decade and will likely play an important role in reshaping health care over the next decade [2,3].

Various specific types of e-mental health services exist, such as online counseling, mental health information websites, self-guided treatment programs, and online support groups. However, those providing a number of core psychological functions (eg, assessment, referral, treatment) are commonly referred to as online or virtual clinics [4-6]. Here, before accessing an online treatment program, some kind of assessment process typically occurs to screen for mental health problems that are less suitable for online therapy, such as imminent risk of suicide, as well as to help ensure the delivery of appropriate treatment of the client’s particular psychological concern [7,8]. Crisis management and/or external referral commonly occur at this stage when the person is at risk / not suitable. The assessment is then generally followed by access to a structured treatment program that often incorporates cognitive behavioral therapy (CBT) techniques because of the established efficacy of this therapeutic modality [9-11]. Once the treatment is completed, a posttreatment assessment is scheduled. Therefore, it is reasonable to consider attrition and its predictors at each 1 of the 3 treatment cycle stages: pretreatment attrition, during treatment attrition, and posttreatment attrition. Examination of attrition and its predictors at each stage would help with early identification of those at risk of dropping out. Being able to do so may result in developing better designs and more targeted interventions to reduce dropouts or noncompletions.

Numerous articles have highlighted the increasing popularity and the rapid growth of online interventions, (eg, [6,8,12-14]) and have discussed the effectiveness of online programs for the treatment of anxiety disorders (eg, [2,6,15-19]). However, many trials are typically small to moderate in sample size (see [16,20]) and include relatively high rates of attrition [12,21,22]; this continues to be a limiting and challenging factor at each stage in the process. Although many papers indirectly provide the specific study dropout rates at the various stages of their study via flowcharts, attrition is generally discussed as a single category (ie, combining pretreatment assessment, during treatment and posttreatment assessment / follow-up attrition together). Similarly, most of the research published to date includes little or no analysis of predictors of attrition at the different stages in the study process, especially pretreatment attrition predictors or attrition bias and its impact on treatment efficacy. Instead, conservative intention-to-treat analyses, which are likely to attenuate differences [16], have been performed to evaluate the efficacy of online treatments, usually with last observation carried forward (LOCF) imputation for attrition cases [23] or the more advanced method of multiple imputation [24-26]. The scope to which intention-to-treat has been used in online treatment research is not clear, but it is recommended and widely used in most published large-scale studies [20]. However, if it can be shown that attrition bias is unlikely, then completer analysis is likely to be a reasonable and accurate approach. Furthermore, if it can be shown that attrition bias is unlikely and if measures are available on more than 2 occasions, a more accurate maximum likelihood longitudinal analysis would be possible [27,28] and highly recommended [29].

Attrition bias notwithstanding, many reviews and discussion papers firmly support the efficacy of e-mental health programs for multiple psychological concerns, such as anxiety disorders, depression, alcohol and other drug problems, and even when these disorders coexist as comorbid conditions (eg, [8,30]). Andersson et al [31] concluded that existing research supported the efficacy of online programs for the treatment of anxiety disorders, especially when a form of CBT framework was used coupled with some client-therapist contact. Amstadter et al [32] found similar results and concluded that common CBT techniques, such as cognitive restructuring and relapse prevention, can be adapted to online programs with relative ease. More recently, Andersson and Titov [6] concluded that evidence supporting the efficacy of self-help Internet-delivered CBT was strong and consistent.

In summary, online therapy is becoming more popular and more studies are attesting to its utilization and efficacy for a variety of mental health issues and disorders. Most online treatment programs consist of structured modules that include CBT techniques because of its established efficacy. Although several studies have examined predictors of attrition for online therapy, studies looking at the predictors at specific treatment time points (pretreatment, during, posttreatment attrition) are very limited. There is also a lack of studies that include analysis of attrition bias and its impact on treatment efficacy.

**Figure 1:**
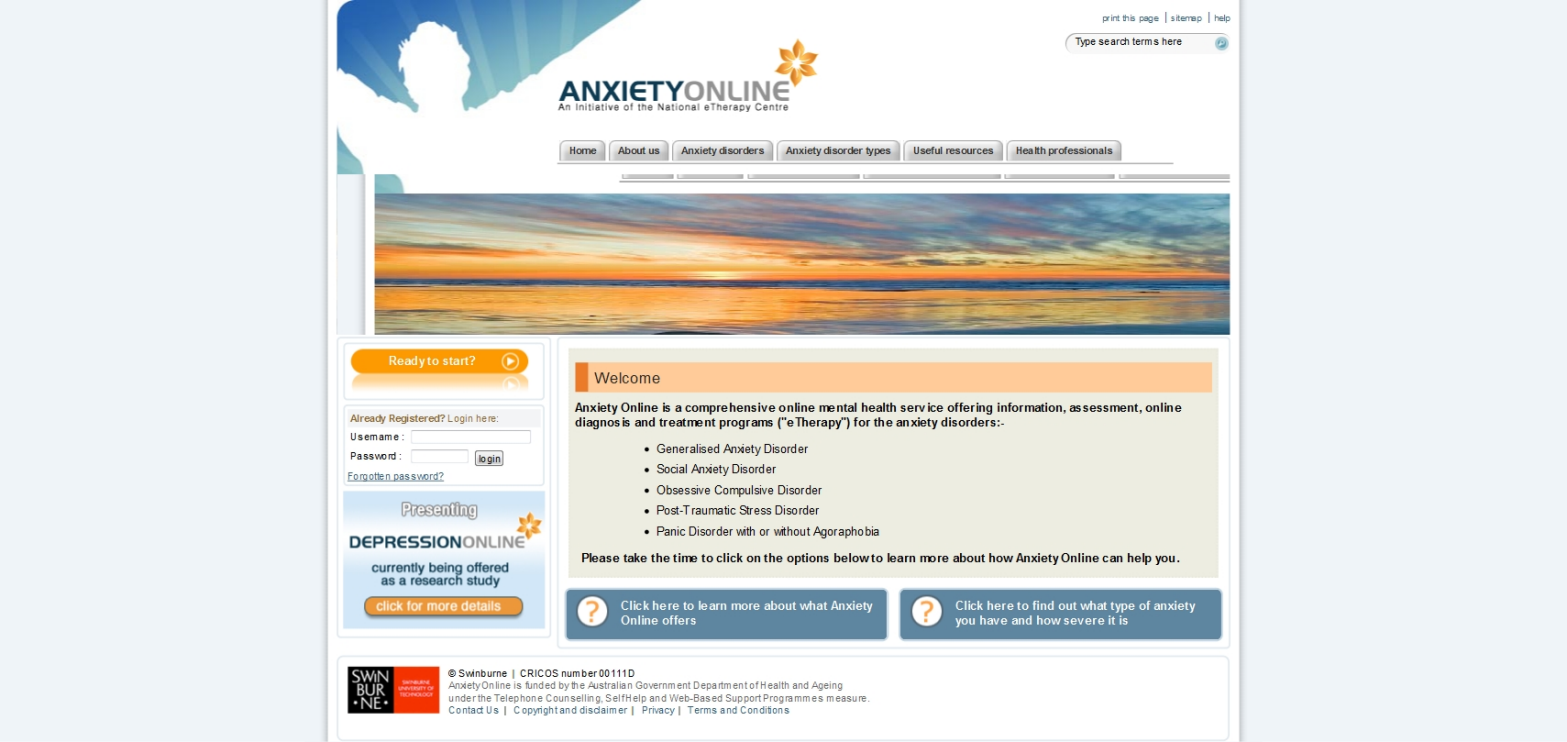
Anxiety Online homepage image.

In this study, posttreatment assessment analysis of attrition and its predictors and the treatment efficacy of the Anxiety Online programs were examined. Identifying the characteristics of those who completed the posttreatment assessment measures will assist in intervening early and in devising ways and directing attention to those who do not complete the posttreatment assessment measures. Anxiety Online is an open-access virtual clinic providing online assessment and diagnosis of 21 mental health disorders defined by the *Diagnostic and Statistical Manual of Mental Disorders* (Fourth Edition, Text Revision; *DSM-IV-TR*) and self-help and therapist-assisted treatment programs for the 5 anxiety disorders (Figure 1) [33]. The Anxiety Online platform was upgraded in September 2013 and now uses the name Mental Health Online [33].

Previously, we examined pretreatment attrition and during treatment formal withdrawal and their predictors in the Anxiety Online data [34]. In this study, we reported on 24 demographic and personal variables and 1 treatment variable that were potentially associated with posttreatment attrition. The impact of attrition bias on the treatment outcome measures was assessed by using the data collected on the 5 fully automated self-help online programs from October 2009 to January 2012. When attrition bias was found to be unlikely, a completer analysis of treatment efficacy was performed based on the pre- and posttreatment treatment outcome measures.

## Methods

### Procedure

The Anxiety Online platform consists of 4 centers: psychoeducational, assessment, treatment, and health care professional training. The psychoeducational center is a website that provides psychoeducational information about prevalence, symptoms, and treatment of anxiety disorders as well as links to useful websites. The assessment center contains electronic psychological assessment screening system (e-PASS) that consists of a demographic/personal questionnaire and the online diagnostic program (together called *assessment measures* henceforth). As shown in Multimedia Appendix 1, the demographic/personal questionnaire contains a total of 24 demographic and personal variables. After completing the questionnaire, a person then completes the e-PASS that consists of more than 100 diagnostic questions, including the Kessler-6 [35] and items that screen for suicide risk and psychosis (see [36] for details). The treatment center provides and manages the 5 anxiety disorder-specific treatment programs. The training center provides the online therapist training programs and the health care practitioner portal. Individuals can access the Anxiety Online service from anywhere in the world via an Internet connection. People complete the e-PASS if they are interested in psychological assessment function and/or if they are interested in online treatment programs. Based on an individual’s response to some of the questions of the e-PASS, a person may be given a primary diagnosis and/or multiple secondary diagnoses in accordance with *DSM-IV-TR* criteria. Those adults (aged 18 years or older) who receive a primary or secondary diagnosis of panic disorder with or without agoraphobia (PD/A), social anxiety disorder (SAD), posttraumatic stress disorder (PTSD), generalized anxiety disorder (GAD), or obsessive-compulsive disorder (OCD) are offered an online 12-week self-help or therapist-assisted treatment program (the therapist-assisted program is only available to Australian residents). Once participants are enrolled into 1 of the 5 fully automated 12-week self-help treatment programs, they cannot enroll in another online program; however, they can opt out of the treatment program by using the “opt out” option available within the program. Those participants who do not opt out are sent automated emails, with several reminders over a 3-week period following their 12-week treatment cycle, asking them to complete the posttreatment assessment measures. The posttreatment measures are essentially the same as the pretreatment measures. Participants are encouraged to complete the e-PASS annually for 5 years following treatment program cycle completion. Those who want to undertake e-PASS are first required to register and consent to the Anxiety Online terms and conditions [33]. The procedures for collecting and reporting of the Anxiety Online data were approved by the Swinburne University Human Research Ethics Committee. From the time of its launch to the public in October 2009 until January 2012, the e-PASS program has been accessed by 10,745 people.

### Treatment Outcome Measures

We identified 6 outcome measures that may be used to indicate successful treatment. The first and second outcome measures were the severity of anxiety disorder--specific symptoms and the severity of major depressive episode (MDE). The disorder-specific severity score is the average of the scores on 6 questions measured on a 8-point Likert scale that assess the level of distress and how much the symptoms of a given disorder interfere in one’s life (see [36]). A reduction in the severity score would suggest a positive treatment outcome. The third outcome measure was the number of diagnosed primary and/or secondary disorders. Based on the individual’s responses to the e-PASS, each person was given 1 primary disorder and 1 or more secondary disorders if warranted. A reduction in the number of diagnosed disorders (called *number of disorders* henceforth) was indicative of a successful treatment outcome. The fourth outcome measure was the total score on the Kessler-6. The Kessler-6 consists of 6 items measured on a 5-point Likert scale measuring nonspecific psychological distress over the past 30 days. Normative data indicate that 71.7% of the population report low distress scores of 6 to 11, 16.6% of the population report moderate distress scores of 12 to 15, 7.16% of the population report high distress scores of 16 to 19, whereas 2.5% of the population report very high distress scores of 20 to 30 [35,37]. A reduction in the Kessler-6 total score would also suggest a successful treatment outcome. The fifth outcome measure was the individual’s self-confidence in managing one’s mental health issues. This self-confidence measure is a self-report question measured on a 5-point Likert scale as shown in Multimedia Appendix 1. An increase in reported self-confidence is suggestive of a positive treatment outcome. The final outcome measure was the individual’s perceived overall quality of life. The quality-of-life measure is a self-report question measured on a 5-point Likert scale as shown in Multimedia Appendix 1. Similar to self-confidence, an increase in the reported overall quality of life was indicative of a positive treatment outcome.

### Participants

**Figure 2:**
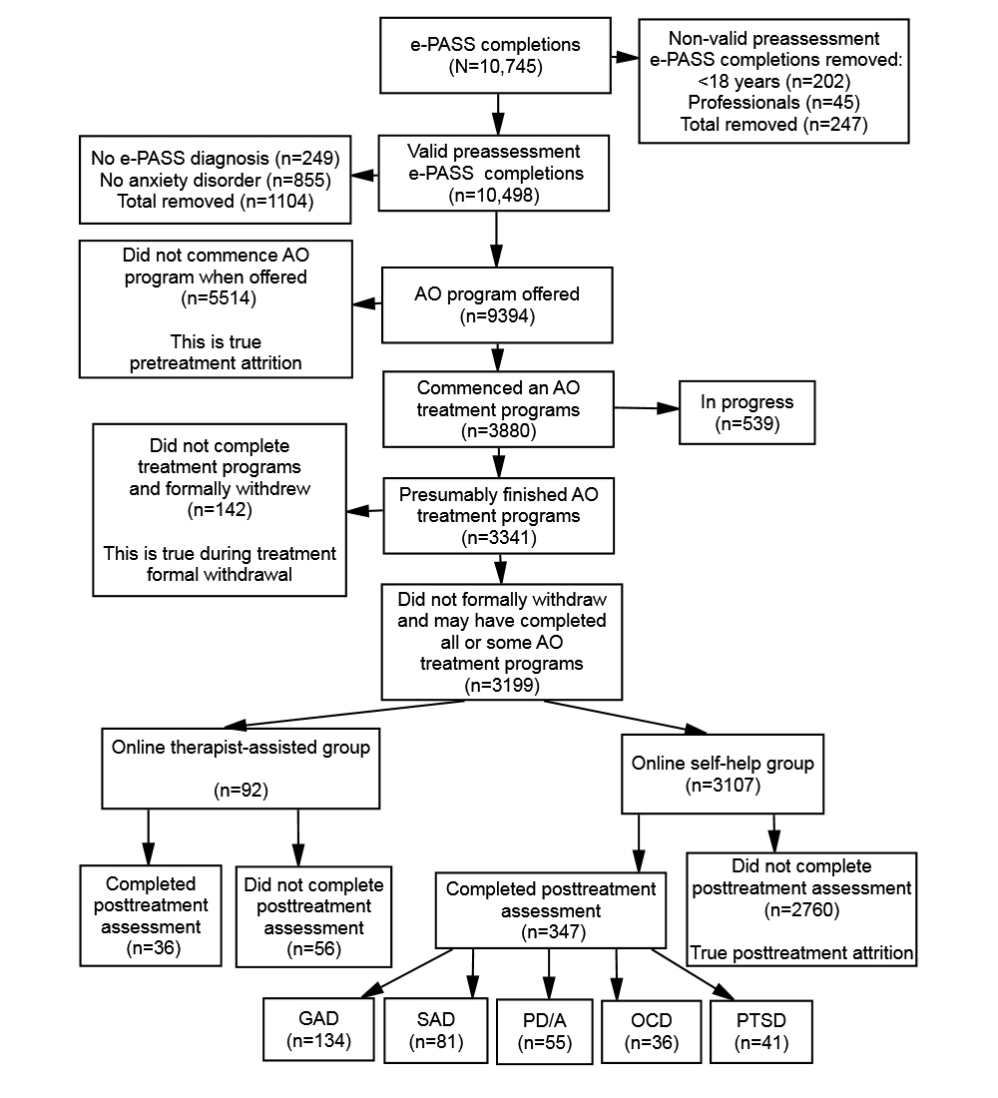
Recruitment and enrollment rate throughout the process.

As shown in Figure 2, a total of 10,745 individuals completed the pretreatment assessment measures between October 2009 and January 2012. Some of those individuals were people younger than age 18 years (n=202) and some were professionals (n=45) who were exploring the assessment instrument. These 247 individuals were removed from the data leaving 10,498 valid completers of the e-PASS program. In addition, another 249 individuals who did not receive an e-PASS diagnosis of any of the 21 disorders and another 855 individuals who did not receive an e-PASS primary or secondary diagnosis of any of the 5 anxiety disorders were also removed. The removal of these individuals resulted in a sample consisting of 4771 (50.79%) individuals whose primary diagnosis and 4623 (49.21%) individuals whose secondary diagnosis was 1 of the 5 anxiety disorders, for a total of 9394 e-PASS pretreatment completers. All 9394 were offered a treatment program, although it was recommended that those with a primary diagnosis other than anxiety should seek help elsewhere. Only 3880 individuals accepted and commenced a 12-week online treatment, whereas 5514 individuals did not accept the offer of an online treatment program. At the time of analysis, there were 539 individuals still undergoing treatment; 3199 individuals had not formally withdrawn from their treatment program cycle, whereas 142 individuals formally withdrew during their treatment program cycle.

Unfortunately, of the 3199 individuals, only 383 (11.97%) individuals in total completed the posttreatment assessment measures, whereas 2816 (88.03%) individuals did not complete. Of the 3199, a total of 92 (2.88%) individuals selected the therapist-assisted therapy (36 individuals completed the posttreatment assessment measures and 56 did not complete), whereas 3017 (97.12%) individuals selected 1 of the self-help online programs (347 individuals completed the posttreatment assessment measures and 2760 did not complete). To keep the focus on self-help and exclude any therapist intervention, for the purpose of this analysis, we only considered the 347 individuals who selected the self-help online treatment programs and completed the posttreatment assessment measures in comparison with the 2760 individuals who also selected self-help online treatment programs but did not complete the posttreatment assessment measures. The distribution of individuals who enrolled in the 5 online treatment programs and whether they completed or did not complete the posttreatment assessment measures is shown in Table 1.

The self-help online noncompleter group consisted of 860 males whose age ranged between 18 and 78 years with a mean of 37.74 (SD 12.05) years and 1900 females whose age ranged between 18 and 81 years with a mean of 35.11 (SD 11.57) years. The males group (n=860) consisted of 614 (71.4%) males who reported living in metropolitan areas, 176 (20.5%) males in regional areas, 63 (7.3%) males in rural areas, and 7 (0.8%) males who reported living in remote areas. The females group (n=1900) consisted of 1204 (63.37%) females who reported living in metropolitan areas, 447 (23.53%) females in regional areas, 230 (12.11%) females in rural areas, and 19 (0.01%) females who reported living in remote areas.

The self-help online completer group consisted of 117 males whose age ranged between 19 and 75 years with a mean of 42.19 (SD 12.72) years and 230 females whose age ranged between 18 and 75 years with a mean of 40.34 (SD 12.68) years. The males group (n=117) consisted of 76 (65.0%) males who reported living in metropolitan areas, 31 (26.5%) males in regional areas, 9 (7.7%) males in rural areas, and 1 (0.9%) male who reported living in remote areas. The females group (n=230) consisted of 144 (62.6%) females who reported living in metropolitan areas, 59 (25.7%) females in regional areas, 26 (11.3%) females in rural areas, and 1 (0.4%) female who reported living in remote areas.

The first part of the analysis investigated the differences between the posttreatment assessment completers and noncompleters for the 5 anxiety treatment programs for the Kessler-6 total score and the demographic and personal variables shown in Multimedia Appendix 1. Posttreatment attrition is defined as the ratio of posttreatment assessment noncompleters to the total number of participants, and it is this measure and its predictors that are the focus of this study. Because of the large number of individuals who chose not to complete the posttreatment assessment measures, the second part of the analysis investigated the potential impact of posttreatment attrition bias on the treatment outcome measures of the 5 fully self-help online treatment programs. By using G*Power 3.1 [38], a minimum of 34 participants in each of the 5 anxiety treatment programs were needed to achieve a power of 80% (α=.05) to detect a moderate effect size of 0.5 on treatment outcomes. The number of participants in the 5 treatment programs ranged from 36 to 134 participants, so the interpretation of these results should be considered reliable.

**Table 1:** Number of individuals enrolled in the self-help online programs that completed or did not complete the posttreatment assessment measures.

Anxiety online programs	Self-help, n (%)		Total
	Completed posttreatment assessment	Did not complete posttreatment assessment	
GAD	134 (11.57)	1024 (88.43)	1158
SAD	81 (9.56)	766 (90.44)	847
PD/A	55 (9.95)	498 (90.05)	553
PTSD	41 (13.76)	257 (86.24)	298
OCD	36 (14.34)	215 (85.66)	251
Total	347 (11.17)	2760 (88.83)	3107

### Analysis

The initial univariate analysis used chi-square tests of association to determine which of the pretreatment assessment demographic and personal variables had a significant relationship with posttreatment attrition. A multivariate analysis was then used to confirm the univariate results. Multivariate binary logistic regression analysis with a forward selection approach was performed to identify the significant predictor variables. The final model was evaluated using a Hosmer-Lemeshow test.

To assess attrition bias, Heckman’s 3-step method was used [39]. The first step involves using the attrition demographic and personal predictor variables in a binary logistic regression to predict the probability that each participant will complete the posttreatment assessment measures. The second step involves using this predicted probability to compute the Mills ratio for each participant using the ratio of the normal probability and cumulative distribution function for the residuals. The third step involves determining the effect of (predicted) attrition risk on the change in treatment outcome measures between pre- and posttreatment. This is accomplished by constructing a multivariate general linear model based on the difference between pre- and posttreatment treatment outcome measures and treatment programs with the Mills ratio entered as a covariate. A nonsignificant Mills ratio effect and a nonsignificant Mills ratio by programs interaction effect are indicative of no attrition bias. That is, noncompleters of posttreatment assessment measures would have given similar answers to those given by completers.

Finally, repeated measures MANOVA followed by repeated measures ANOVAs for the treatment outcome measures (anxiety-specific severity rating, MDE severity rating, Kessler-6 total score, number of disorders, self-confidence, and quality of life) were used to evaluate the 5 self-help online treatment programs separately. The data supported the assumptions of normality and homogeneity. The Cohen’s *d* effect size classification scheme (0.20 for small effect, 0.50 for moderate effect, and 0.80 for large effect) was used [40]. SPSS version 20 (IBM Corp, Armonk, NY, USA) was used to produce all results.

## Results

### Posttreatment Attrition (Profile of Posttreatment Assessment Completers)

As shown in Table 1, most participants (2760/3107, 88.83%) enrolled in 1 of the 5 self-help online treatment programs, did not formally opt out, but did not complete the posttreatment assessment measures. Only 347 participants completed the posttreatment assessment measures yielding a posttreatment assessment completion rate of 11.17% (347/3107) and a posttreatment attrition rate of 88.83% (2760/3107).

As shown in Table 2, the chi-square tests of association and *F* tests showed 9 variables that were significantly associated with completing the posttreatment assessment measures. Those who completed the posttreatment assessment measures tended to differ from those who did not complete posttreatment assessment measures in several ways. On average, it was more likely that the completers of posttreatment assessment measures had heard about the program through traditional media rather than the Internet, were seeking online assistance with the primary goal of finding a self-help program; were willing to provide consumer feedback, were receiving mental health assistance, were nonsmokers, rated their self-confidence as “good,” said that they learned best by reading, had a slightly lower pretreatment Kessler-6 total score, and were older in age.

**Table 2:** Predictor analysis for attrition categories for posttreatment assessment completers and noncompleters for online self-help group (N=3107).

Variables		Attrition categories		Test of association		
		Completers (n=347)	Noncompleters (n=2760)	χ^2^ (df)	*F*_1, 3105_	*P*
**How did you hear about us?, n (%)**				25.6 (4)		.001
	Internet	108 (31.1)	1205 (43.66)			
	Health professional	58 (16.7)	407 (14.75)			
	Friend/family	20 (5.8)	198 (7.17)			
	Traditional media	104 (20.0)	623 (22.57)			
	Other	57 (16.4)	327 (11.85)			
**Reason for seeking online assistance, n (%)**				13.0 (1)		.001
	To complete 1 of the self-help programs	231 (66.6)	1557 (56.41)			
Provide consumer feedback (yes), n (%)		182 52.5	1285 (46.56)	8.6 (1)		.01
Currently receiving mental health assistance (yes), n (%)		146 (42.1)	986 (35.72)	5.4 (1)		.02
Do you smoke? (yes), n (%)		41 (11.8)	464 (16.81)	5.7 (1)		.02
**Self-confidence, n (%)**				13.3 (4)		.01
	Very poor	15 (4.3)	193 (6.99)			
	Poor	76 (21.9)	716 (25.94)			
	Neither	132 (38.0)	1049 (38.01)			
	Good	114 (32.9)	693 (25.11)			
	Very good	10 (2.9)	109 (3.95)			
**How do you best learn?, n (%)**				13.6 (3)		.004
	Hearing	25 (7.2)	151 (5.47)			
	Reading	125 (36.0)	811 (29.38)			
	Looking and watching	43 (12.4)	522 (18.91)			
	Doing	154 (44.4)	1276 (46.23)			
Pre–Kessler-6 (total score), mean (SD)		15.97 (4.90)	16.99 (4.80)		13.73	.001
Age (years), mean (SD)		40.97 (12.71)	35.93 (11.79)		55.29	.001

As shown in Table 3, the final binary logistic regression with forward selection of predictors for posttreatment attrition contained 6 significant predictors. The Hosmer-Lemeshow goodness-of-fit test indicated an adequate model fit. When statistically controlling for the other variables in the model, we found significant odds ratios for the following predictors: how participants first heard about the Anxiety Online program, reason for registering, age, whether currently receiving assistance for mental health concerns, best method of learning, and pre–Kessler-6 total score. We should note that the same predictors were also found to be significantly associated with posttreatment attrition using chi-square tests as shown in Table 2.

The expected odds for completing the posttreatment assessment measures in order of significance were as follows: 3% increase in likelihood for each year increase in age; 3% reduction in likelihood for each additional point an individual scored on the Kessler-6 total score; 1.76 and 1.42 times higher for those who heard about the Anxiety Online from the traditional media (TV, radio, magazine, newspaper) and from friends or family members, respectively, relative to other sources (eg, brochure, mail-out, newsletter, e-bulletin, lecture, conference, support group, through work, Facebook); 1.42 times higher for individuals who gave “seeking to use 1 of the self-help online programs” as a reason for joining the program relative to all other reasons; 1.94 and 1.76 times higher for those indicating that they learn best by hearing or reading, respectively, relative to those who said they learn best by looking or watching; and 1.40 times higher for those who reported that they were receiving mental health assistance relative to those who were not receiving mental health assistance.

**Table 3:** Binary logistic regression model for posttreatment assessment attrition.

Variables		Wald (df)	*P*	OR (95% CI)
**Heard (reference group: other sources)**		11.79 (4)	.02	
	Internet	3.30 (1)	.07	1.38 (0.98-1.96)
	Health professional	0.13 (1)	.72	1.10 (0.66-1.82)
	Friend or family	5.06 (1)	.02	1.42 (1.05-1.92)
	Traditional media	9.92 (1)	.002	1.76 (1.24-2.49)
Reason (online self-help)(reference group: other reasons)		8.20 (1)	.004	1.42 (1.12-1.81)
Age		33.93 (1)	.001	1.03 (1.02-1.04)
Currently receiving mental health assistance (reference group: none)		7.33 (1)	.007	1.40 (1.10-1.78)
**Learning (reference group: looking/watching)**		11.09 (3)	.01	
	By hearing	5.95 (1)	.02	1.94 (1.14-3.32)
	By reading	9.03 (1)	.003	1.76 (1.22-2.55)
	By doing	3.37 (1)	.07	1.40 (0.98-2.00)
Pre–Kessler-6 total score		6.64 (1)	.01	0.97 (0.94-0.99)
Constant		100.15 (1)	.001	0.03

### Attrition Bias and Evaluation of Treatment Outcomes

Six personal and demographic variables were found to be significantly associated with posttreatment attrition: age, Kessler-6 total score, “how did you first hear about the Anxiety Online?,” reason for registering, “how do you best learn?,” and whether the person was currently receiving mental health assistance. These 6 variables were used in a binary logistic regression to predict the attrition category for all participants. A probability estimate of completing the posttreatment measures was calculated for each participant who actually completed the posttreatment assessment measures. The Mills ratios for all 347 completers of posttreatment assessment measures were calculated using Heckman’s method [39].

Next, the difference between the pretreatment score and the posttreatment score on the 10 treatment outcome measures (5 severity scores for each anxiety disorder, MDE severity score, Kessler-6 total score, number of disorders, self-confidence, and quality of life) for each participant was calculated. To analyze attrition bias for the 347 clients who selected 1 of the online self-help anxiety treatment programs, a MANOVA analysis was carried out to compare the improvement in the 10 treatment outcome measures (represented by the differences in scores of these outcome measures at pre- and posttreatment) for the 5 treatment programs with Mills ratio included as a covariate. Results revealed that the Mills ratio had no significant effect (*F*_10, 328_=0.550, *P*=.85) and that there was no significant interaction effect between the Mills ratio and the programs (*F*_40, 1246_=0.683, *P*=.93). Therefore, these results suggested that there was unlikely to be any attrition bias for all 5 fully automated anxiety treatment programs, allowing completer analysis to be used.

### Analysis of Treatment Outcomes

#### Overview

A repeated measures MANOVA for each fully automated self-help online treatment program was carried out with the 6 treatment outcome measures (Kessler-6 total score, number of disorders, self-confidence, quality of life, MDE severity, and the anxiety-specific severity measure) followed up by repeated measures ANOVAs with analysis of effect size using Cohen’s *d*. To arrive at more conservative and more accurate values for the effect size, the correlations (*r*) between the pre- and posttreatment outcome measures (reported in Table 4) were ignored in the calculation of Cohen’s *d* and its 95% CI [41]. Results of these analyses, means, standard deviations, and other parameters are displayed in Table 4.

**Table 4:** Summary of means, standard deviations, correlations for pre- and posttreatment results, *F* values, *P* value, and Cohen’s *d* and its 95% CI for treatment outcome measures.

Treatment outcome measures			Pretreatment, mean (SD)	Posttreatment, mean (SD)	*F* (*df*)	*P*	*r*	Cohen’s *d* (95% CI)
**GAD (n=134)**								
		GAD severity	3.28 (1.54)	2.00 (1.67)	85.35 (1,133)	.001	.50	0.80 (0.54, 1.08)
		MDE severity	2.07 (2.07)	1.23 (1.89)	28.36 (1,133)	.001	.57	0.42 (0.07, 0.74)
		Kessler-6	16.59 (4.57)	13.62 (4.30)	99.89 (1,133)	.001	.70	0.67 (–0.10, 1.40)
		# of disorders	4.31 (1.98)	3.46 (2.18)	34.40 (1,133)	.001	.68	0.41 (0.07, 0.78)
		Self-confidence	3.07 (0.88)	3.63 (0.82)	49.40 (1,133)	.001	.41	–0.66 (–0.81, –0.52)
		Quality of life	3.43 (0.82)	3.66 (0.82)	10.86 (1,133)	.001	.51	–0.28 (–0.42, –0.14)
**SAD (n=81)**								
		SAD severity	3.12 (1.63)	1.95 (1.87)	37.17 (1,80)	.001	.52	0.67 (0.31, 1.08)
		MDE severity	1.60 (1.99)	1.46 (2.17)	0.29 (1,80)	.59	.37	0.07 (–0.37, 0.54)
		Kessler-6	15.59 (4.97)	13.72 (4.86)	12.34 (1,80)	.001	.52	0.38 (–0.70, 1.44)
		# of disorders	4.46 (2.16)	3.60 (2.15)	17.53 (1,80)	.001	.64	0.40 (–0.07, 0.87)
		Self-confidence	3.02 (0.88)	3.47 (0.92)	12.55 (1,80)	.001	.22	–0.50 (-0.69, –0.30)
		Quality of life	3.32 (0.91)	3.54 (0.90)	5.71 (1,80)	.02	.57	–0.24 (–0.44, –0.05)
**OCD (n=36)**								
		OCD severity	2.81 (1.87)	1.97 (2.37)	5.25 (1,35)	.03	.48	0.40 (–0.21, 1.17)
		MDE severity	1.76 (2.41)	1.04 (1.95)	6.50 (1,35)	.02	.72	0.33 (–0.46, 0.97)
		Kessler-6	14.42 (5.60)	13.28 (5.62)	2.67 (1,35)	.11	.72	0.20 (–1.63, 2.04)
		# of disorders	4.33 (2.15)	3.03 (2.25)	15.16 (1,35)	.001	.58	0.59 (–0.11, 1.33)
		Self-confidence	3.25 (0.91)	3.81 (0.82)	12.59 (1,35)	.001	.41	–0.65 (–0.94, –0.38)
		Quality of life	3.58 (0.87)	3.75 (1.03)	2.69 (1,35)	.11	.81	–0.18 (-0.46, –0.16)
**PD/A (n=55)**								
	PD/A severity		3.19 (1.87)	1.51 (2.14)	35.41 (1,54)	.001	.46	0.84 (0.34, 1.40)
	MDE severity		1.75 (2.05)	1.15 (2.01)	7.41 (1,54)	.009	.67	0.30 (–0.25, 0.83)
	Kessler-6		14.89 (4.69)	12.51 (4.69)	22.86 (1,54)	.001	.69	0.51 (–0.73, 1.75)
	# of disorders		4.62 (2.16)	3.44 (2.52)	14.91 (1,54)	.001	.54	0.50 (–0.07, 1.17)
	Self-confidence		3.05 (1.03)	3.55 (0.90)	18.01 (1,54)	.001	.61	–0.52 (–0.79, –0.28)
	Quality of life		3.56 (1.15)	3.67 (0.94)	1.29 (1,54)	.26	.79	–0.11 (–0.41, 0.14)
**PTSD (n=41)**								
	PTSD severity		3.01 (1.74)	2.09 (1.99)	7.21 (1,40)	.01	.32	0.50 (–0.04, 1.10)
	MDE severity		2.42 (2.16)	1.65 (2.10)	5.64 (1,40)	.02	.53	0.36 (–0.30, 1.00)
	Kessler-6		17.51 (4.93)	14.73 (5.23)	9.19 (1,40)	.004	.33	0.55 (–0.96, 2.15)
	# of disorders		5.39 (2.97)	4.32 (3.07)	8.63 (1,40)	.005	.70	0.35 (–0.55, 1.29)
	Self-confidence		3.12 (0.95)	3.76 (0.89)	22.35 (1,40)	.001	.57	–0.70 (–0.99, –0.42)
	Quality of life		3.10 (0.83)	3.49 (0.90)	15.85 (1,40)	.001	.74	–0.45 (–0.70, –0.18)

#### Generalized Anxiety Disorder Treatment Program

A significant multivariate time effect was found for the GAD program (*F*_6, 128_=26.85, *P*<.001). Subsequent repeated measures ANOVAs showed significant improvements on all 6 treatment outcome measures. GAD severity produced a large effect size, Kessler-6 and self-confidence produced moderate effect sizes, and MDE severity, number of disorders, and quality of life produced small effect sizes.

#### Posttraumatic Stress Disorder Treatment Program

A significant multivariate time effect was found for the PTSD program (*F*_6, 35_=4.45, *P*=.002). Subsequent repeated measures ANOVAs showed significant improvements on all 6 treatment outcome measures. Self-confidence, Kessler-6, and PTSD severity produced moderate effect sizes, whereas quality of life, MDE severity, and number of disorders produced small effect sizes.

#### Social Anxiety Disorder Treatment Program

A significant multivariate time effect was found for the SAD program (*F*_6, 75_=8.36, *P*<.001). Subsequent repeated measures ANOVAs showed significant improvements on 5 of the 6 treatment outcome measures. SAD severity and self-confidence produced moderate effect sizes, whereas Kessler-6, number of disorders, and quality of life produced small effect sizes. MDE severity produced a very small and nonsignificant effect size.

#### Panic Disorder With or Without Agoraphobia Treatment Program

A significant multivariate time effect was found for the PD/A program (*F*_6, 49_=8.89, *P*<.001). Subsequent repeated measures ANOVAs showed significant improvements on 5 of the 6 treatment outcome measures. PD/A severity produced a large effect size, whereas Kessler-6, self-confidence, number of disorders, and MDE severity produced moderate effect sizes. Quality of life produced a small nonsignificant effect size.

#### Obsessive-Compulsive Disorder Treatment Program

A significant multivariate time effect was found for the OCD program (*F*_6, 30_=4.18, *P*=.004). Subsequent repeated measures ANOVAs showed significant improvements on 4 of the 6 treatment outcome measures. Self-confidence and number of disorders produced moderate effect sizes, whereas OCD severity and MDE severity produced small but significant effect sizes. Kessler-6 and quality of life produced small nonsignificant effect sizes.

## Discussion

The purpose of this study was to examine posttreatment assessment attrition and its predictors, and to assess the potential for attrition bias and its impact on treatment outcome measures for the Anxiety Online self-help programs. The posttreatment assessment attrition rate for the self-help programs was found to be 89%. This is a large posttreatment assessment attrition rate compared with therapist-assisted randomized controlled trials of online treatment (eg, 13% [42], 6.4% [43], and 0% [44]). However, our attrition rate compares favorably with attrition rates reported by similar open-access fully automated treatment programs (eg, [21]).

The e-PASS program collected data on 24 demographic and personal variables and 1 measure of psychological distress, the Kessler-6. Chi-square tests of association and binary logistic regression were used to relate these variables to posttreatment assessment attrition. Results revealed that the likelihood of completing posttreatment assessment measures declined for participants with a greater Kessler-6 total score and increased for older participants, participants who heard about the program through the traditional media and from family and friends, those who were looking to complete a self-help online program, participants receiving assistance for mental health concerns, and for participants who reported learning best by reading, hearing, and doing rather than looking and watching. Those that joined the program because they wanted to receive online therapy were more likely to complete the posttreatment assessment measures potentially because of greater motivation and commitment to the program and interest around their treatment outcome. Participants who learn best by reading, hearing, and doing would likely be more involved in their learning than those who learn more passively by looking and watching. This difference in the reported learning style between being actively or passively involved may explain why those reporting the former style were more likely than those reporting the latter style to complete the posttreatment assessment measures. Participants who were receiving mental health services were likely to be more invested and actively engaged in managing their mental health and, therefore, they were more likely to show the tendency to complete the posttreatment assessment measures.

Interestingly, older participants were more likely to complete the posttreatment assessment measures, perhaps because with age comes a greater sense of commitment to the task. The age of our sample ranged between 18 and 78 years and because age was not a significant predictor of pretreatment attrition and formal withdrawal during treatment attrition, age was not a discriminatory factor [34]. This is especially encouraging because online programs are often thought to be of greater interest to younger cohorts [45], whereas these results suggest online programs are applicable across the age spectrum.

Similar to traditional face-to-face treatment programs, online programs ideally start with an assessment of the issues, then move to treatment of these issues, and then proceed to assessment of the impact of treatment on these issues. These 3 phases (pretreatment assessment, treatment, and posttreatment assessment) make up the standard design for any treatment program; therefore, attrition and its predictors at these different stages should be examined separately and not viewed as a single category. At each phase, there will be those who will start but not finish. Therefore, it is important to assess not only attrition at each of these 3 phases but also the predictors of attrition. In AL-Asadi et al [34], the predictors of pretreatment attrition and formal withdrawal during treatment were identified. In this study, we identified the predictors of posttreatment attrition. If we were to consider the findings of these 2 studies combined, we discover that there was no single predictor that is present in all phases, that some variables were predictors in only 1 phase, and that only some variables were predictors in 2 phases. For example, we found those who heard about the program from family/friends and traditional media sources, those who were seeking online self-help, those who learn best by reading, and those who had lower levels of psychological distress were more likely to accept and commence treatment and more likely to complete the posttreatment assessment measures. On the other hand, those who were concerned about anxiety, those who reported poor quality of life, and those who were prepared to make or in the process of making changes were more likely to accept and commence treatment and more likely not to formally withdraw from treatment.

By using Heckman’s method [39], attrition bias was assessed to be nonsignificant and consequently it is reasonable to conclude that those who did not complete the posttreatment assessment measures would have responded in the same way as those who completed the posttreatment assessment measures. The nonsignificant attrition bias allowed the use of completer analysis to assess the impact of the treatment programs in place of overconservative intention-to-treat analyses. Analyses of the treatment outcomes revealed that for all 5 treatment programs (GAD, SAD, PD/A, OCD, and PTSD) there was a significant effect in reducing the diagnostic anxiety-specific severity reported by participants, reducing the total number of diagnosed disorders, and increasing the reported self-confidence in dealing with ones’ mental health issues.

The MDE severity scores significantly decreased for participants in 4 of the treatment programs with the SAD group showing a small nonsignificant improvement. This finding that the treatment for anxiety disorder produced not only significant reduction in the severity of the anxiety-specific symptoms but also in the severity of symptoms of depression is indicative of the efficacy of online treatment to provide transdiagnostic treatment. These results are consistent with the conclusions of Andersson and Titov [6] and Johansson et al [46].

Psychological distress, as measured by the Kessler-6 total score, significantly decreased for participants in 4 of the treatment programs with the OCD group showing a small nonsignificant improvement. Similarly, the quality-of-life rating significantly improved for participants in 3 of the treatment programs with the OCD and the PD/A groups showing nonsignificant improvement. Overall, these results support the efficacy of online treatment of the 5 anxiety disorders. Cohen’s *d* within-group treatment effect sizes ranged from 0.40 to 0.84 for the relevant anxiety-specific severity scores. These results are consistent with the typical range of 0.4-0.7 reported for other self-help online programs [36,47,48].

These results suggest that efficacious fully automated self-help online treatment programs for a variety of anxiety disorders can be delivered to anyone with an Internet connection, anywhere, at any time. This increase in accessibility to treatment should make it easier for those whose mobility is restricted, those who feel uncomfortable being seen in a local mental health clinic, those who do not have local resources, and those who are unable to adhere to regular appointments to access mental health treatment. Online programs may have other potential advantages. For example, the potential to reach large and/or rural populations at a fraction of the cost associated with face-to-face therapy, and the privacy and anonymity of accessing therapy in one’s own home reduces the cost as well as the stigmatization [49-51] associated with accessing face-to-face services. Two literature reviews by Musiat and Tarrier [1] and by Lal and Adair [3] agreed that online programs were cost effective, but disagreed on geographical and time flexibility. Musiat and Tarrier [1] concluded that there was limited reporting around the advantages of geographical, time flexibility, and stigma, whereas Lal and Adair [3] concluded that geographical flexibility, timing and convenience, and anonymity were the strengths and benefits associated with e-mental health programs. These differing opinions highlight the need for more research. Another added advantage is that online therapy makes it easy to provide people with several means of presentation of educational material, such as written, video, or audio formats, which can facilitate matching learning methods to preferred learning styles/preferences [3,32]. The mode of online therapy offers a promising and cost-effective alternative to face-to-face therapy [36], especially when considering the underengagement of the public with face-to-face services for mental health concerns [52-54]. This percentage becomes even lower for those who are living far from major urban centers and those who reside in rural communities where concerns over stigma is heightened and access and resources are limited [55,56]. In sum, although evidence is not consistent across all benefits, online therapy remains a potentially viable mode of service delivery that may provide both time- and cost-effective intervention and address obstacles associated with traditional therapy, such as stigma, travel issues, and lengthy waitlists to see a therapist. Therefore, online intervention may provide those who otherwise could not or would not seek out psychological treatment with the opportunity to avoid several limiting obstacles and receive effective therapy.

However, there are 4 major limitations that should be noted. Firstly, Anxiety Online platform (now Mental Health Online) is a cost-free system open to anyone in the world with Internet access. The design of this system does not require a control group; thus, it is difficult to make any conclusion regarding causal relationships between the treatment programs and improvements. Moreover, the lack of a control group and the high rate of posttreatment attrition make any conclusion about the efficacy of this online therapy preliminary. Secondly, the e-PASS uses online assessment procedures exclusively that rely on self-report to determine the diagnoses of participants. The use of automated online assessment for the purpose of assigning diagnoses is a limitation of this study in itself because the reliability of online diagnostic assessment tools has been questioned [6].

Thirdly, a single study that examined the psychometric properties of the e-PASS concluded that the treatment outcome measures have high test-retest reliability and reasonable convergent validity (D Nguyen, unpublished PhD thesis, Swinburne University, 2013). However, the small sample size and some disagreement with structured clinical interviews in terms of the severity levels required for a clinical diagnosis suggest that further validation studies with large sample sizes are needed. Consequently, more validation studies based on the newly released *DSM-5* criteria must be conducted.

Fourthly, the use of completer analysis may overestimate the effectiveness of the treatment programs when attrition bias is suspected. However, in this case, attrition bias was found to be nonsignificant suggesting that the results accurately reflect the true effectiveness of the treatment programs. The use of the more conservative Cohen’s *d* values that do not take correlations into account [41] showed that effect sizes were reasonable despite the use of a conservative effect size measure.

As for the Anxiety Online platform, the high posttreatment attrition rate was a weakness of this platform. It appears that sending several automated email reminders over a 3-week period following the 12-week treatment cycle may be a relatively ineffective way to encourage sufficiently large numbers of people to complete the posttreatment measures. However, we should acknowledge that, although still high, the multiple reminder email reminders may be 1 of the reasons why the Anxiety Online posttreatment attrition rate is slightly lower than other fully automated self-help open-access systems. This is confirmed by the higher posttreatment completion rates for Anxiety Online therapist-assisted program versions (36/92, 39%) involving a weekly email from a trained therapist.

Having participants complete the posttreatment measures is certainly a challenging task. This is probably exacerbated given the participants have already undertaken the assessment measures before treatment and, therefore, they know how demanding the posttreatment assessment will be. Telephone calls after the multiple email reminders may prove useful in further reminding participants, although this would impact on cost and also detracts from the fully automated nature of the system. Alternatively, motivation to complete posttreatment assessment measures may be increased by educating participants on the importance of completing the posttreatment assessment measures to allow the improvement of the treatment programs for future participants. In addition, asking participants to enter into a “behavioral contract” beyond the terms and conditions might be valuable (eg, pledge commitment and completion of modules and posttreatment assessment measures before they can commence).

Research on e-mental health has been taking place over the past decade or so examining its efficacy with a number of different disorders. However, it is important to continue to investigate a broader range of mental health problems, other therapeutic modalities besides CBT, and the issues related to geographic and time flexibility, stigma, and specific populations—especially older adults. Furthermore, and in view of the high attrition rates, especially those with open-access fully automated self-help online programs, it is recommended that when establishing treatment efficacy, researchers should consider examining the question of attrition bias. If attrition bias is found to be nonsignificant, completer analysis or maximum likelihood longitudinal methods should be used to assess treatment accuracy rather than the overly conservative intention-to-treat analyses.

## Multimedia Appendix 1

Self Report Online Questionnaire.

## Abbreviations

CBT: cognitive behavioral therapy
DSM-IV-TR: Diagnostic and Statistical Manual of Mental Disorders (4th Edition, Text Revision)
e-PASS: electronic psychological assessment screening system
GAD: generalized anxiety disorder
MDE: major depressive episode
OCD: obsessive-compulsive disorder
PD/A: panic disorder with or without agoraphobia
PTSD: posttraumatic stress disorder
SAD: social anxiety disorder
