# Supplementary material for: Posttreatment Attrition and Its Predictors, Attrition Bias, and Treatment Efficacy of the Anxiety Online Programs
Source: J Med Internet Res. 2014 Oct 14;16(10):e232. doi: 10.2196/jmir.3513 (PMC4211028; doi:10.2196/jmir.3513)
Supplement: Supplementary file 1 [file jmir_v16i10e232_app1.pdf]

<u>Appendix “1”</u>

Self Report Online Questionnaire

How did you first hear about us (Heard)?

Why are you wishing to register (1-9 reasons) (Reason)?

Would you like to join the research register and receive emails about participating in research?

Would you be willing to provide consumer feedback...?

In what year were you born (Age_years)?

What is your gender (Sex)?

What is your relationship status (Relationship_Status)?

In what type of setting do you live (Setting)?

What is your current employment status (Employment_Status)?

What category best describes your highest schooling (Schooling)?

What is your highest level of post-school/tertiary education (Post-Secondary)?

What is your current main mental health concern (MH_concern)?

Are you currently receiving mental health assistance?

Have you accessed mental health in the last 12 months?

Have you ever accessed mental health services?

Do you have any diagnosed physical health condition?

How many times have you visited a medical doctor in the last month (Doctor_visit)?

Do you smoke?

Do you drink alcohol?

Do you feel that you have adequate social support?

Rate your overall level of self-confidence in managing your mental health (1= very poor to 5=very good)

Rate your overall quality of life (1= very poor to 5=very good)

Are you making changes regarding your mental health (1, 2=not interested, 3=interested, 4=Doing so now)

How do you best learn? (Hearing, Reading, Looking, Doing)
